# Robust Computational Analysis of rRNA Hypervariable Tag Datasets

**DOI:** 10.1371/journal.pone.0015220

**Published:** 2010-12-31

**Authors:** Maksim Sipos, Patricio Jeraldo, Nicholas Chia, Ani Qu, A. Singh Dhillon, Michael E. Konkel, Karen E. Nelson, Bryan A. White, Nigel Goldenfeld

**Affiliations:** 1 Institute for Genomic Biology, University of Illinois at Urbana-Champaign, Urbana, Illinois, United States of America; 2 Loomis Laboratory of Physics, University of Illinois at Urbana-Champaign, Urbana, Illinois, United States of America; 3 Department of Animal Sciences, University of Illinois at Urbana-Champaign, Urbana, Illinois, United States of America; 4 Avian Health and Food Safety Laboratory, Washington State University, Puyallup, Washington, United States of America; 5 School of Molecular Biosciences, Washington State University, Pullman, Washington, United States of America; 6 The J. Craig Venter Institute, Rockville, Maryland, United States of America; BC Centre for Excellence in HIV/AIDS, Canada

## Abstract

Next-generation DNA sequencing is increasingly being utilized to probe microbial communities, such as gastrointestinal microbiomes, where it is important to be able to quantify measures of abundance and diversity. The fragmented nature of the 16S rRNA datasets obtained, coupled with their unprecedented size, has led to the recognition that the results of such analyses are potentially contaminated by a variety of artifacts, both experimental and computational. Here we quantify how multiple alignment and clustering errors contribute to overestimates of abundance and diversity, reflected by incorrect OTU assignment, corrupted phylogenies, inaccurate species diversity estimators, and rank abundance distribution functions. We show that straightforward procedural optimizations, combining preexisting tools, are effective in handling large (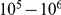) 16S rRNA datasets, and we describe metrics to measure the effectiveness and quality of the estimators obtained. We introduce two metrics to ascertain the quality of clustering of pyrosequenced rRNA data, and show that complete linkage clustering greatly outperforms other widely used methods.

## Introduction

There is a long history of using environmental 16S rRNA [1] to estimate microbial diversity [2]. While early techniques relied on using clone libraries [3], next-generation high-throughput sequencing technology, such as pyrosequencing, directly generates vast libraries of sequences [4]. Next-generation high-throughput sequencers are capable of producing large datasets of more than a million reads from a single plate [5]. As the size of these datasets grow, the ability to computationally manage and characterize such data becomes a larger and more critical component of microbial ecology.

The goal of analyzing these sequences is to quantify the diversity and abundance distributions of organisms present in the environment. As pyrosequencing technology advances, our ability to measure microbial diversity increases. Already, this technique has been used to study the diversity of microbiomes from a variety of environments [6], [7], resulting in reports of a so-called “rare biosphere” of low-abundance organisms [8].

In order to assess the microbial diversity present in any dataset, the ability to appropriately measure the distance between different sequences and to reliably group them into operational taxonomic units (OTUs) is paramount. Typically, the abundance of OTUs is plotted in a rank abundance plot. These plots have been used as a gold standard for ecological population modeling for many decades [9]. In addition, the OTU groupings are utilized by other metrics in determining relative species compositions, microbial diversity, and community comparisons [10], [11]. While much effort and controversy has been focused on measurements of the quality of next-generation sequences [8], [12]–[15], or the interpretation of pyrosequencing flowgrams [16], less attention has been given to computational analysis of pyrosequenced 16S rRNA data after quality processing, despite the large discrepancies in OTU numbers and diversity when different analysis methods are used [14], [16]–[18].

There are two major components to the analysis of OTU abundance. The first is multiple alignment of 16S rRNA or fragments of 16S rRNA. The second is clustering the sequences based on a distance metric. The purpose of this paper is to provide a careful discussion of the computational analysis of alignment and clustering of pyrosequencing datasets, identifying sources of error, and appropriate ways to handle the data to mitigate these artifacts. In particular, we use the Calinski-Harabasz (CH) index [19] to compare the quality of clustering, and we find unexpectedly large differences in the performance of different algorithms. We show that data analysis is surprisingly sensitive to even small errors in multiple alignment and clustering, but that with relatively little difficulty, these artifacts can be substantially mitigated using a judicious combination of preexisting tools, and others that we have made available on the Web (http://tornado.igb.uiuc.edu). Following these procedures results in a robust characterization of microbial ecosystems.

### Multiple Alignment: NAST and Infernal

Multiple alignment is the starting point of almost all analyses performed on microbiome sequences. Most phylogeny [20], [21], community distance estimates [11], [22], and abundance distributions [23]–[25] ultimately rely on input from a multiple alignment to compute sequence distances within a consistent alignment template.

The goal of multiple alignment is to align sequences according to their evolutionary relationships. In order for a multiple alignment to be meaningful in this context, all sequences in the multiple alignment must have a common origin. The various match, mismatch, and indel events then represent possible reconstructions of the evolution of those related sequences. In contrast to pairwise alignment, multiple alignment leverages conserved features of an entire gene family to obtain a broader evolutionary picture. This picture can then be fed into various algorithms such as maximum-likelihood phylogeny [20], [21], [26] in order to reconstruct the evolutionary relationships between the individual sequences.

The use of 16S rRNA sequences for discerning evolutionary relationships has a long history. The very first studies that organized the Bacteria according to their evolutionary relationships and resulted in the discovery of the Archaea utilized this important ribosomal molecule as a molecular fossil [27] and it still remains the most widely used evolutionary marker in microbial ecology today [5], [8], [28]. As such, it is not surprising that a number of tools exist which are specifically tailored to 16S rRNA such as the NAST pipeline [29] or Ribosomal Database Project [30].

These specialized 16S rRNA alignment tools all incorporate information about the 16S rRNA secondary structure. The importance of the secondary structure is two-fold. First, the conservation of 16S rRNA sequences stems from the conserved structure. Second, unlike proteins which are built from up to 20 different amino acids, there are only 4 basic RNA bases. Randomly chosen RNA bases have a greater chance of aligning well with one another than randomly chosen protein sequences, making it more difficult to distinguish between evolutionary relationships and random matches. Secondary structure can, and should, be used to provide extra discriminatory power beyond that available from the one-dimensional sequence alone.

The NAST algorithm [29] tries to align new sequences against a precalculated multiple alignment template, and has been integrated into many commonly used 16S rRNA analysis tools such as Mothur [11] and GreenGenes [31]. Typically, this template is hand-curated to include the appropriate secondary structure considerations. In this paper we will use the SILVA SEED SSURef database version 102 [32] as the template and refer to this alignment method as NAST+SILVA. The weaknesses of this method are that errors in the hand-curated multiple alignment propagate and that alignment against a fixed-size template necessitates the inclusion of purposeful misalignments. Overall, this results in alignments that are sometimes inconsistent with alignments based on secondary structure. An example of this is shown in the alignments in Figure S2b. By contrast, Infernal [33], which has been integrated into the Ribosomal Database Project 16S rRNA Pipeline [30], aligns sequences against a predefined structure. However, even among the well-conserved structures of 16S rRNA, there exist hypervariable regions which vary in their secondary structure from taxon to taxon. These regions cannot be aligned to a fixed structural template, and are left unaligned by Infernal (leading to a multiple alignment whose length is not fixed but may be different in different datasets). An example of this Infernal's alignment is shown in Figure S2a. It is important to note that while both methods align to a seed model of some sort, the practical difference is that RDP+Infernal do better in regions of strong secondary structure whereas NAST+SILVA do better in hypervariable regions.

To exploit this distinction, one can merge the best alignments from each tool by combining the hypervariable regions aligned using the NAST algorithm with the regions of strong secondary structure aligned by Infernal. An example of sequences aligned using the merging method is given in Figure S2c. One can also make adjustment to the multiple alignment by hand. Done properly, this can produce a better quality alignment than automated methods alone. An example of the merged alignment in which the hypervariable region was further hand-curated is given in Figure S2d. For a brief description of the process and the tools that we developed to perform the merging and hand-curation, please refer to the Materials and Methods section.

With the availability of a variety of tools that perform multiple sequence alignment, it is imperative to have a way to assess its quality. One way to do that is through maximum-likelihood (ML) phylogeny. ML phylogeny tries to identify the set of relationships with the best likelihood value. Conversely, ML scores can also be used to judge the likelihood of a multiple alignment reflecting sequence evolution. Indeed, similar measures have been used in the past [34] and tools such as SATÉ [35] already take advantage of this measure when iterating between multiple alignments and phylogeny to automate the search for the best alignment and tree. However, exploring enough multiple alignments for large datasets is prohibitively expensive and therefore remains impractical for now. Nonetheless, it is feasible to use the ML method in order to compare the quality of alignments by measuring their likelihood values, and we use this below to compare different alignment strategies.

### Clustering Algorithms

Clustering algorithms, such as complete linkage [36], are essential for quantifying the diversity of microbial communities. The goal of clustering is to group sequences that are within some measure of evolutionary distance. Distances can be calculated using many different metrics such as percent sequence identity (PSI) or distance along the phylogenetic tree branches. Ideally, a clustering algorithm should identify the natural boundaries between the clusters without utilizing more clusters than necessary to account for the entire dataset. Ultimately, clustering should accurately reflect the underlying phylogenetic and taxonomic distribution of sequences.

Complete linkage clustering (as implemented by Mothur [11]) has become the most widely-used clustering algorithm in microbial ecology. It relies on input from a distance matrix that can be generated from the pairwise distances between sequences in a multiple alignment. When calculating sequence distances, it is important to clearly note how alignment gaps are dealt with. One can ignore the gaps (like Phylip DNADIST does [25]) or count them in a number of different ways [23]. Once pairwise distances are obtained, complete linkage operates by progressively merging smaller clusters into larger ones, as long as each element in a cluster is within a defined distance from other elements in the cluster [36].

The performance of linkage clustering algorithms, for example as implemented in Mothur, scales poorly (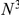, where 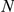 is the number of sequence reads) as the number of sequence reads generated per study increases. In the studies reported below, we reimplemented Mothur's clustering algorithm, achieving an improvement in computational complexity (scaling as 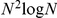), better memory usage, and an overall speedup that is typically a factor of 5–10, leading to the ability to handle datasets with 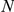 up to about 30,000. Despite these improvements, it is understandable that heuristic, computationally efficient algorithms have been developed, such as FastGroup [37] and ESPRIT [38].

**Figure 1:**
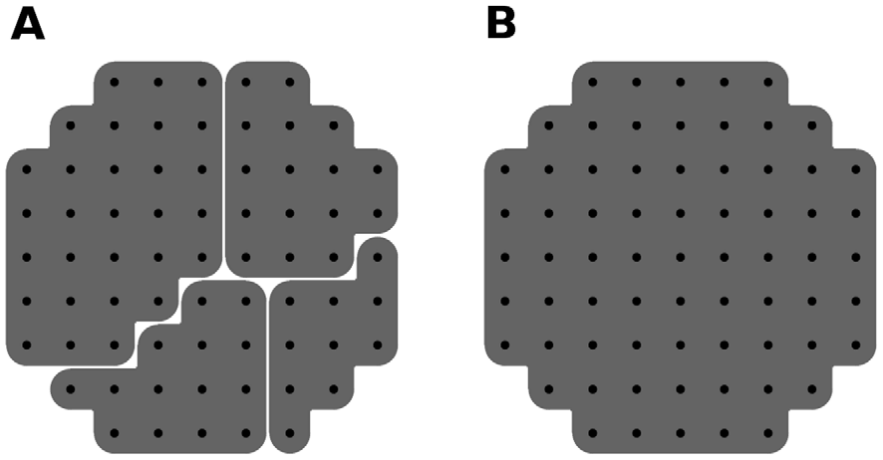
Calculation of clustering a set of points in a plane. (a) FastGroup's method. (b) complete linkage clustering. Both of these clusterings are performed with the same radius 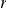 equal to the radius of the set of points. FastGroup constructs 4 clusters whereas complete linkage finds 1.

FastGroup does not order clustering in any particular way, but instead chooses a sequence at random, grouping everything within a defined PSI distance of that sequence. As an example of how that is different from the complete linkage clustering employed by Mothur, consider the clustering of a scatter of points in two dimensions, as shown in Figure 1. The two-dimensional space is a very simple example of sequence space, with position in the space corresponding to the particular sequence of an organism. A set of points in this space, if sufficiently close to one another, represents a set of sequences that can be considered to be grouped into a single equivalence class—in other words, an OTU. The largest allowable distance between points in a single equivalence class corresponds to the sequence similarity required for sequences to be included in the same OTU (typically 97% is used).

When the FastGroup algorithm is used to group these sequences, with a radius equal to the radius of the circle of points, the number of clustered OTUs can vary, depending on the order of chosen cluster centers. One example of FastGroup's clustering is given in Figure 1a. On the other hand, complete linkage clustering with the same diameter correctly identifies the existence of 1 cluster (Figure 1b), by progressively merging clusters as long as they are within a cluster diameter (see Figure S3 for the progress of the complete linkage algorithm).

ESPRIT [38] goes one step further and does away with multiple alignment entirely and processes the clusters in two steps: the first relying on a k-mer heuristic, similar to that used in BLAST [39], in order to group closely related sequences under one representative sequence; the second relying on pairwise distances between representatives in order to determine the final clusters. Both FastGroup and ESPRIT differ from the more controlled calculations of the complete linkage algorithm, but at the same time promise less computationally intensive results. Before pursuing such alternatives, it is important to understand the differences between the results produced by each of these algorithms.

In other words, do the heuristic algorithms produce natural cluster borders and correct cluster compositions? A natural cluster should have a representative sequence that is near the center of the cluster, i.e. the representative sequence should be one that shares the most similarity to all other sequences in the cluster. Natural clusters should not partition the dataset into more groups than necessary. One way to quantify this goodness of clustering is via the Calinski-Harabasz (CH) index [19] that has been found to be the best in a comprehensive study of 30 different clustering quality indices [40]. In essence, the Calinski-Harabasz index is higher when the cluster centers are further away from each other (i.e. the clusters are better delineated from each other), and when the cluster radii are smaller (i.e. the clusters are tighter). The CH index is also correctly normalized so as to be comparable for different number of OTUs.

## Results

In this work, we demonstrate that different methodologies can lead to very different estimates of OTU abundances. We characterize these differences and deconstruct their two primary sources: multiple alignment and the clustering method used. We measure the performance of both components of this process, restricting ourselves to 16S rRNA based techniques. We also provide metrics to quantitatively evaluate the effectiveness of algorithms used. Our analysis includes an examination of the robustness of these algorithms on real biological data. We perform our analysis on a dataset of 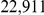 bacterial 16S rRNA sequences (V3 region) with an average length of 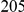
bp from a sample of a chicken caecum. We note however, that our methodology is also applicable to longer 16S rRNA reads.

Before further analysis, we treated our dataset in the following way. To handle length variation among sequences, we trimmed our sequences to only be between the first and last conserved columns in the NAST [29] alignment to SILVA database [32]. We further removed any sequences less than 100 bp long and any sequences that contained an unknown nucleotide (N). After cleanup our dataset had 21,646 sequences.

### Multiple Alignment: Performance

We compared the effectiveness of the different alignment algorithms by using the likelihood values returned by maximum-likelihood phylogeny. In alignment of nucleotide sequences with secondary structure the aligners that are aware of the secondary structure generally outperform those that rely on sequence data alone [41], such as ClustalW [42] and MUSCLE [43]. In addition, these aligners scale poorly with dataset size [44]. Thus, we test the two commonly used 16S rRNA alignment algorithms: RDP [30]+Infernal and NAST in conjunction with the SILVA database [32]. Using ML phylogeny, we find log-likelihood scores of 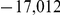 and 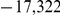 for RDP+Infernal and NAST+SILVA, respectively, as obtained from the FastTree ML algorithm [20]. The merged alignment of RDP+Infernal with NAST+SILVA, described in the introduction, has a log-likelihood value of 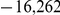, representing an improvement over either of the two algorithms alone. When we perform further hand-curation of hypervariable regions of the 16S V3 in the merged alignment, we obtain a log-likelihood score of 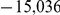
—reflecting the misalignments that can occur in the automated procedure.

We can also take these different multiple alignments and cluster them in order to see how the OTU abundance results depend on the multiple alignment procedure. The OTU numbers after complete linkage clustering with radii 3%, 5% and 7% on seven different alignments are shown in Table 1. Here, “merge” refers to the merging of RDP+Infernal with NAST+SILVA. Note that running the aligners on sequences after quality processing produces thousands of OTUs. However, performing hand-trimming of sequence tails reduces the number of OTUs by an order of magnitude. This suggests that poorly curated alignments may overestimate microbial diversity.

**Table 1:** Dependence of the number of OTUs on the alignment method used. The percentages indicate clustering radius.

Alignment method	Number of OTUs		
	3%	5%	7%
NAST+SILVA (on raw)	1141	646	406
RDP+Infernal (on raw)	3588	2313	1743
Merged (on raw)	3647	2297	1682
NAST+SILVA (on trimmed)	425	251	187
RDP+Infernal (on trimmed)	406	234	169
Merged (on trimmed)	393	227	165
Hand-curated	354	207	153

Trimmed sequences refer to sequences in which elementary hand-curation was performed (see introductory paragraphs of Results for more information). Merged refers to the multiple alignment that is a merging of the hypervariable regions aligned by NAST+SILVA regions with strong secondary structure conservation aligned by RDP+Infernal. See Introduction for more information. Note that crude hand-curation can reduce numbers of OTUs by a whole order of magnitude.

### Clustering: Performance

**Figure 2:**
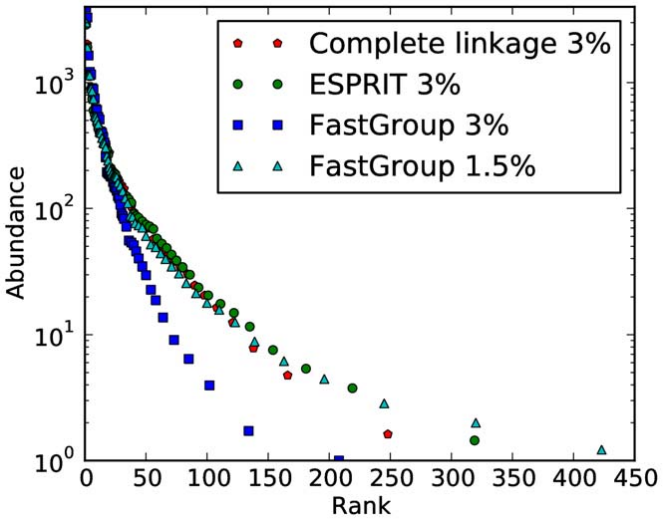
Rank abundance curves obtained with different algorithms and/or clustering distances. Notice that FastGroup with 1.5% sequence distance identifies a similar rank abundance curve to those of ESPRIT and complete linkage. However, it is not evident from the Figure that FastGroup identifies almost two times the number of OTUs than ESPRIT or complete linkage.

We compared the three clustering algorithms (complete linkage, FastGroup and ESPRIT) by running them on the hand curated alignment described in the previous section. We can visualize the effect of the choice of clustering algorithm by comparing rank abundance curves and cluster compositions. Rank abundance curves for the chicken caecum dataset are compared in Figure 2, for the 3% sequence difference clustering distance (and 1.5% FastGroup). As demonstrated by the curve, complete linkage clustering, ESPRIT and FastGroup at 1.5% obtain the same shape of the curve, but FastGroup aw 3% finds a very different one. This is because complete linkage at distance 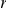 corresponds to clusters where every element is at distance 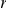 to every other element in the cluster. On the other hand, FastGroup guarantees that every element is only at distance 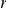 from the chosen center of the cluster. This means that there may be elements in the same cluster that are at a distance of 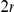 from each other. Hence, 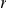 for FastGroup denotes the “radius” of the cluster, whereas 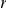 for complete linkage denotes the “diameter” of the cluster. Thus, FastGroup at 1.5% sequence distance can be compared to complete linkage and ESPRIT at 3%.

We find that FastGroup at 1.5% overestimates the number of OTUs in the sample. The binning in Figure 2 hides the fact that the number of OTUs found by FastGroup at 1.5% is much larger than that of ESPRIT and complete linkage. FastGroup at 1.5% finds 834 OTUs compared to complete linkage (354) and ESPRIT (434). Most of these extra OTUs are singletons. Of the 834 FastGroup OTUs, 440 are singleton OTUs. In comparison, complete linkage has 103 OTUs that are singletons out of total of 354. ESPRIT has 122 OTUs that are singletons out of 434 total. This is in accordance to the idea that is sketched in Figure 1, that a clustering algorithm such as FastGroup overestimates the number of OTUs.

We now evaluate the clustering quality via the CH index. For 3% clustering distance, complete linkage has a CH index of 167,771, whereas ESPRIT clustering has a CH index of 244. FastGroup with 1.5% clustering radius has CH index of 94,696. We note that complete linkage significantly outperforms other linkage clustering algorithms: nearest neighbor linkage (single linkage) got a CH index of 14,042 and average neighbor linkage got 23,512. We can also compare CH indices for clustering assignments that have roughly the same number of OTUs, rather than the same clustering distance. We find that complete linkage has CH indices between 140,000 and 160,000 for a range of clustering assignments with 200 to 300 OTUs. ESPRIT produced two clustering assignments in this range: first with 235 OTUs has a CH score of 280, and second with 303 OTUs has a CH score of 286. Finally, FastGroup (with 3% distance) got a CH score of 16,000 for a clustering assignment with 251 OTUs.

Another way to quantitatively judge the goodness of clustering is by comparing the OTU assignments to the structure of the maximum likelihood phylogenetic tree. To do this, we count the number of clades in a phylogenetic tree that contain only sequences of the same OTU (as determined by the clustering algorithm). We expect that a good clustering assignment will have many such clades. Two examples of this calculation are sketched out in Figure S4. We ran this calculation on 2 phylogenetic trees, one made by FastTree [20] (FT) and one made by RAxML [21] (RX), both inferred from our dataset described above. We find that complete linkage clustering has the most clades with uniform OTUs: 863 in FT and 698 in RX. Clustering with FastGroup (with 1.5% distance), we find 427 clades in FT and 367 in RX, whereas ESPRIT performs the most poorly: 6 clades in FT and 7 in RX.

**Figure 3:**
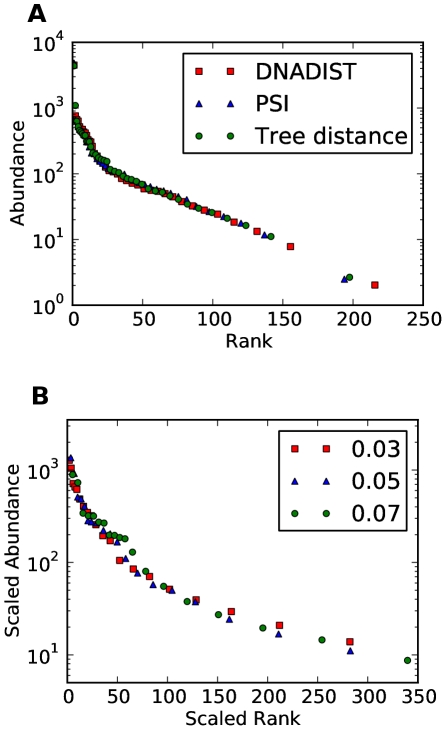
Two checks that should be used to verify quality of rank abundance curves. Both plots show rank abundance curves of the chicken caecum dataset. (a) Comparison of rank abundance curves for three clusterings using three different distance metrics. We compare the clusterings that produce 300 OTUs (which corresponds to different radii 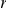 for different metrics). (b) Rank abundance curve is robust if it does not change shape (functional form) when a different clustering radius is used. The rank abundance curves for different clustering radii all fall onto the same curve after rescaling the ranks to the same number of OTUs (while keeping area under the curve constant).

We also explored if the rank abundance curves depend upon the clustering distance metric used. We find that the complete linkage clustering with the hand curated multiple alignment is very robust with respect to the choice of distance metric. In Figure 3a we compare the rank abundance curves (made by complete linkage) for three different distance metrics: Phylip DNADIST [25], percent sequence identity and distance along phylogenetic tree constructed by FastTree. We see that regardless of the choice of the distance metric, the shape of the rank abundance curve is conserved.

If we seek universal laws in the rank abundance data, we should expect that the shape of the rank abundance curve does not depend upon the particular clustering radius chosen. If instead rank abundance changes significantly with radius, that would imply that there is an interesting interplay between population dynamics and sequence distance. The complete linkage clustering with our hand curated multiple alignment is found to be robust with respect to choice of clustering radius. As an example, see Figure 3b for the rank abundance curves of our chicken caecum microbial sample clustered at three different distances. By rescaling the axis of the rank abundance curves, while keeping areas under them constant, we can compare the functional forms (i.e. shapes) of the rank abundance curves. The figure shows that the chicken caecum microbial sample rank abundance seems to obey a universal law over a range of clustering distances.

## Discussion

In the literature, the quality of data from pyrosequencing has been called into question [17], [45], especially with regard to its use in surveys of OTU diversity. Concern has been directed mostly at the experimental process of acquiring DNA sequences with high quality. Sogin *et al.* [8] showed that a number of heuristics can guarantee that per-base error rate of pyrosequencing is lower than that of Sanger sequencing while retaining more than 90% of data. Other artifacts that raised concern came from the shortness of pyrosequenced reads [12], [13]. Quince *et al.* [16] showed that reinterpreting pyrosequencing flowgrams via a maximum-likelihood scheme can lead to fewer OTUs. In this paper we showed that a significant part of the discrepancy may arise from different computational analyses employed. Recent work [18] that has been similarly motivated has been commensurate with the conclusion that clustering is an important step in OTU analysis. In particular, they suggest that a preclustering step can help fix problems where deep sequencing overestimates species richness. Our work presents more general quantitative metrics that can be used as a standard for clustering programs. In addition, we find that calculating the log-likelihood of a maximum-likelihood phylogenetic tree is a good way to compare the quality of nucleotide alignments. Clustering quality index such as Colinski-Harabasz can even be used to verify what clustering radius is appropriate for a particular dataset. Our results that the multiple alignment and distance metrics can have a large effect on OTU abundances are also in agreement with recent work by Schloss [46].

In general, we found that multiple alignments can have a large influence on OTU abundance information, and the automated 16S rRNA alignment tools should be subjected to hand curation. Fast clustering tools such as ESPRIT do not make use of a multiple alignment and rely on k-mer heuristics to calculate pairwise distances between ungapped sequences. Our results show that such tools, intended to improve upon complete linkage, actually perform significantly worse. Hence, even with increasing dataset sizes, it is important to verify that the clustering method used performs no worse than complete linkage. We developed tools that ease the burden of performing hand curation and complete linkage of large contemporary datasets. These are available as supplementary software and are described in more detail in Materials and Methods.

Finally, we summarize for the reader's convenience, step-by-step recommendations for handling a large 16S rRNA dataset, based on the analyses we have reported here. These are graphically illustrated in Figure S1.

1. *Quality Processing:* Remove short reads and sequences with unknown nucleotides (N). Make an alignment to the SILVA database [32], via NAST [29] as implemented by Mothur [11]. Trim sequences to be between the first and last strongly conserved columns in this alignment.
2. *Alignment:* From the trimmed dataset, produce another alignment through RDP pipeline's [30] front end to the Infernal aligner [33]. Merge the two alignments (NAST+SILVA with RDP+Infernal) using the tool that's a part of the TORNADO pipeline at http://tornado.igb.uiuc.edu. Further hand-optimize hypervariable regions of the reads by using the tool available on the website above.
3. *Cluster:* Cluster the dataset using the complete linkage tool available on the website above.

Further analysis can be performed by calculating estimators in Mothur [11], or by estimating phylogenetic trees via RAxML [21] or FastTree [20].

## Materials and Methods

### V3 rRNA amplicon sequencing

We used the V3 rRNA sequences from the chicken caecum from batch B of a previous study [47]. PCR specific primers flanking the V3 hypervariable region of bacterial 16S rRNA were used to generate PCR products for pyrosequence analysis. The forward fusion primers for pyrosequencing included 454 Life Sciences A adapter, and barcode 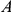 fused to the 5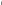 end of the V3 primer 341F (5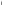 gcctccctcgcgccatcag-ACGAGTGCGT-CCTACGGAGGCAGCAG3 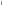) or with barcode 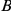 (5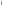 gcctccctcgcgccatcag-ACGCTCGACA-CCTACGGAGGCAGCAG3 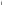). The reverse fusion primer included 454 Life Sciences B adapter fused to 5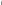 end of V3 primer 534R (5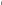 gccttgccagcccgctcag- ATTACCGCGGCTGCTGG3 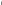). Cycling conditions (20 cycles) were; initial denaturation at 94

C for 5 min; 20 cycles of 94

C 30 s, 60

C 30 s and 72

C 30 s; then 72

C 7 min for final extension. The amplicon products were cleaned using PCR purification clean-up kit and SPRI size exclusion beads. The quality of products was assessed using a Bioanalyzer using DNA1000 chip. The fragments in amplicon libraries were subjected to a single pyrosequence run using a 454 Life Science Genome Sequencer GS FLX (Roy J. Carver Biotechnology Center, University of Illinois). The resulting dataset had 22953 sequences of average length 204.7 bp. Before further analysis was performed, we performed basic filtering. We removed all sequences that were shorter than 100 bp reducing the number of sequences to 21646. The sequences have been uploaded to GenBank (accession numbers HQ293272-HQ315544).

### Multiple Alignments

We compared 4 different alignment methods as illustrated in Figure S2. (1) We fed the sequences into Infernal [33] with bacterial secondary structure template as provided by RDP [30]. (2) We aligned the sequences to the SILVA database [32] using the NAST [29] algorithm as implemented by Mothur [11], [48] (align.seqs command). (3) The results of (1) and (2) were then fed into a merger script we have made available on the Web at http://tornado.igb.uiuc.edu/. (4) The merged data sets' hypervariable regions were then hand curated using splicer, a tool we developed and made available on the Web as part of our pipeline TORNADO at http://tornado.igb.uiuc.edu/. This tool allowed us to greatly reduce the number of unique snippets of the hypervariable region of V3 down from 21,646 to about 200, by cutting the longest hypervariable subregion from the alignment, and then dereplicating it. These snippets of sequences in the hypervariable subregion ranged from 1 bp to about 30 bp. This meant that we only needed to hand-curate 200 short snippets to handle the alignment of the hypervariable region. These snippets were separated into two groups according to their secondary structure: loop, and stem-loop-stem. We used RNAfold web server [49]–[52] to verify the structure. The two groups were then hand curated and merged back into the complete multiple alignment using the splicer merge command. For clarity, the process of using splicer is described in Figure S5. All multiple alignments are available at http://tornado.igb.uiuc.edu/.

### Likelihood Scores

Each data set described in the previous section was dereplicated producing 2215 clones each. Likelihood scores were then computed for each dataset using FastTree 2.1.1 with command line parameters -gamma -nt -gtr.

### Distance metrics

We compared 3 different distance metrics to generate Figure 3a. (1) Phylip DNADIST 3.67 [25] with default model parameters. (2) Percent sequence difference calculated using a program we developed, psi-distance, available at http://tornado.igb.uiuc.edu/. The program constructs pairwise differences by calculating the number of letters that are different between every two sequence (gap is considered a letter). This number is then divided by the average of the ungapped lengths of the two sequences compared [23]. (3) Tree distance calculated from the phylogenetic tree calculated by FastTree in the previous section. The tree distances were acquired by calculating tree branch lengths from the Newick formatted tree using the tree-distance program we developed, available at http://tornado.igb.uiuc.edu/.

### Clustering algorithm

Three different clustering algorithms were compared, all on the hand-curated dataset. (1) Complete linkage clustering with furthest neighbors, as implemented in c-linkage, a program we developed that is available at http://tornado.igb.uiuc.edu/. We tested that the program produces the same results as Mothur, but much faster and with less memory usage since it works in 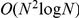. For a comparison of running times of c-linkage and Mothur version 1.12.3, when clustering up to a clustering cutoff of 10% see Figure S6. (2) FastGroup [37] with no trimming, PSI difference of 97% with gaps. (3) ESPRIT [38], for which the dataset was first degapped.

### Cluster Metric

We evaluated the quality of the clustering by calculating the Calinski-Harabasz index [19], [40], [53]. The implementation of the program that calculates the index is available at http://tornado.igb.uiuc.edu/.

## Supporting Information

Figure S1 **Diagram of our proposed 16S rRNA alignment pipeline, TORNADO.** After the preliminary clean up step, we align the sequences in two different ways. First, we use Mothur [11] to align our sequences to the SILVA [32] database. Second, we align using Ribosomal Database Project's front end [30] to the Infernal aligner [33]. We then merge the two, using Infernal's secondary-structure-aware alignments and SILVA's alignment of hypervariable region. Finally, we manually curate the hypervariable regions, using a helper tool, splicer, we developed (see Fig. S5).
(TIFF)

Figure S2 **Snippets of 9 reads aligned using the 4 different methods described in this paper.** The 9 reads are of the V3 region of the 16S rRNA. (**a**) Sequences aligned via RDP [30] which uses the Infernal aligner [33]. Note that the hypervariable region is left unaligned (bases 36 through 64). (**b**) Sequences aligned via NAST [29] (as implemented by Mothur [11]) to the SILVA [32] database. Notice the inconsistencies in the alignment of the regions with strong secondary structure conservation (bases 5, 25, 29, 72 through 79, and 85). (**c**) Sequences aligned using the merge program in the tool we developed, TORNADO (http://tornado.igb.uiuc.edu). The merge process takes the unaligned, hypervariable parts of the sequence aligned by (a) and replaces them by the alignment in (b). (**d**) Sequences aligned like in (c), but with the final hand-curation step of the hypervariable regions.
(TIFF)

Figure S3
**Illustration of the process of the complete linkage algorithm.** Smaller clusters are progressively merged into larger ones as long as no two elements of a cluster are farther than 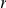 from each other.(TIFF)

Figure S4 **Sketch of the calculation of the number of clades with uniform OTUs.** A phylogenetic tree with 2 different cluster (OTU) assignments is shown. The cluster assignment is indicated by OTU number and color. Both cluster assignments have 2 uniform clades (interior nodes indicated by +1). (**a**) The uniform clades are: one made up of two OTU 1 organisms, and one made up of three OTU 3 organisms. (**b**) The uniform clades are: one made up of two OTU 1 organisms and one made up of three OTU 1 organisms.
(TIFF)

Figure S5 **Using splicer, a part of the TORNADO pipeline, to perform hand curation.** Dereplicating the hypervariable region significantly reduces the effective number of snippets of sequences one needs to hand curate (4 instead of 6 in this example). In our dataset of around 20,000 sequences, there were only around 200 unique sequence snippets in the hypervariable region varying in length between 1 and 30 bp.
(TIFF)

Figure S6 **Comparison of running times of c-linkage with the running times of Mothur.** The two programs were benchmarked on artificial datasets of 1000, 2000, 4000, 6000 and 8000 elements. The sripts used to generate these datasets and run the benchmarks are available at http://tornado.igb.uiuc.edu.
(TIFF)
