# Supplementary figures and images for: Robust Computational Analysis of rRNA Hypervariable Tag Datasets
Source: PLoS One. 2010 Dec 31;5(12):e15220. doi: 10.1371/journal.pone.0015220 (PMC3013109; doi:10.1371/journal.pone.0015220)

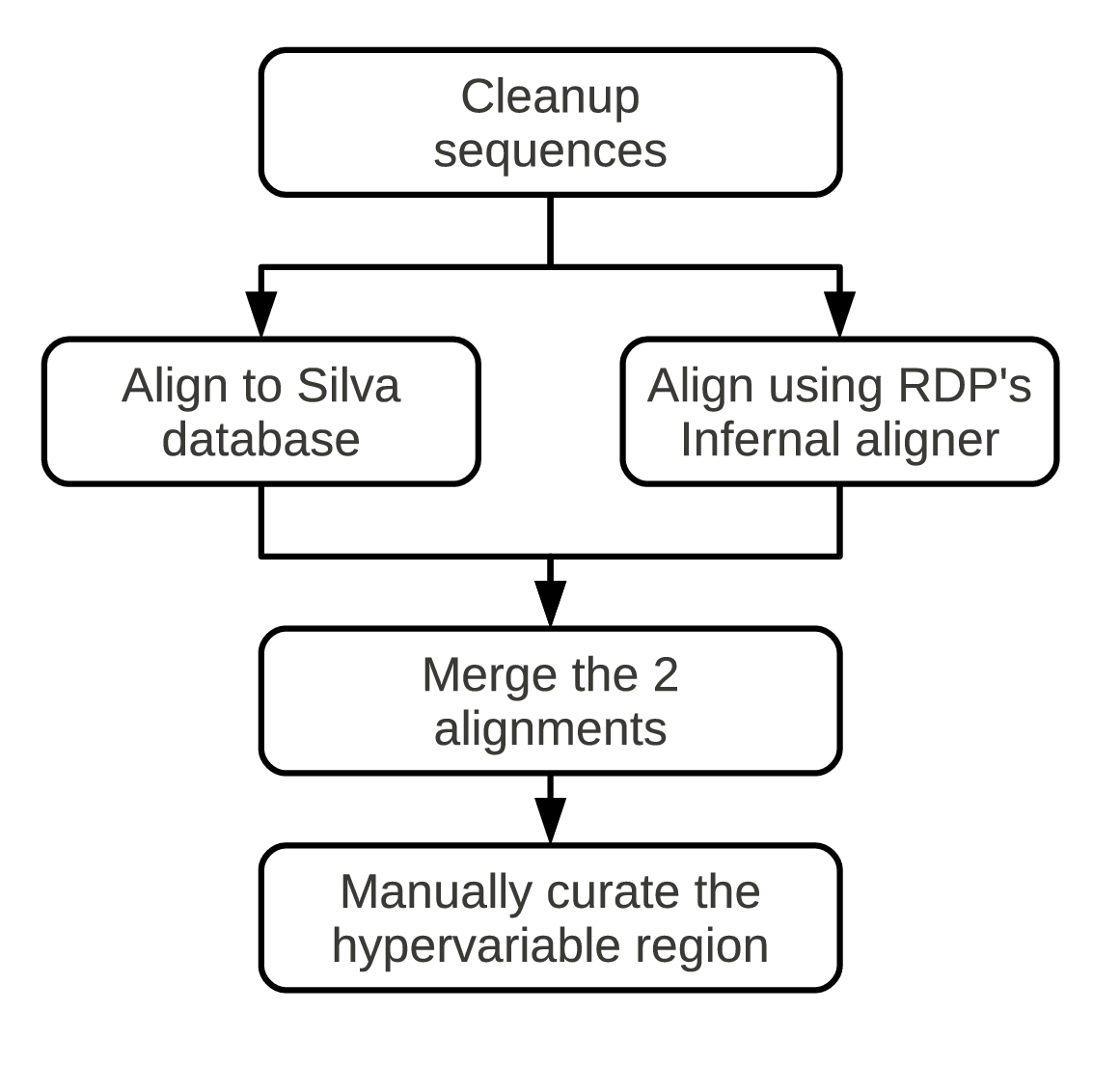

Supplement: Figure S1 — Diagram of our proposed 16S rRNA alignment pipeline, TORNADO. After the preliminary clean up step, we align the sequences in two different ways. First, we use Mothur [11] to align our sequences to the SILVA [32] database. Second, we align using Ribosomal Database Project's front end [30] to the Infernal aligner [33]. We then merge the two, using Infernal's secondary-structure-aware alignments and SILVA's alignment of hypervariable region. Finally, we manually curate the hypervariable regions, using a helper tool, splicer, we developed (see Fig. S5). (TIFF) [file pone.0015220.s001.tiff]

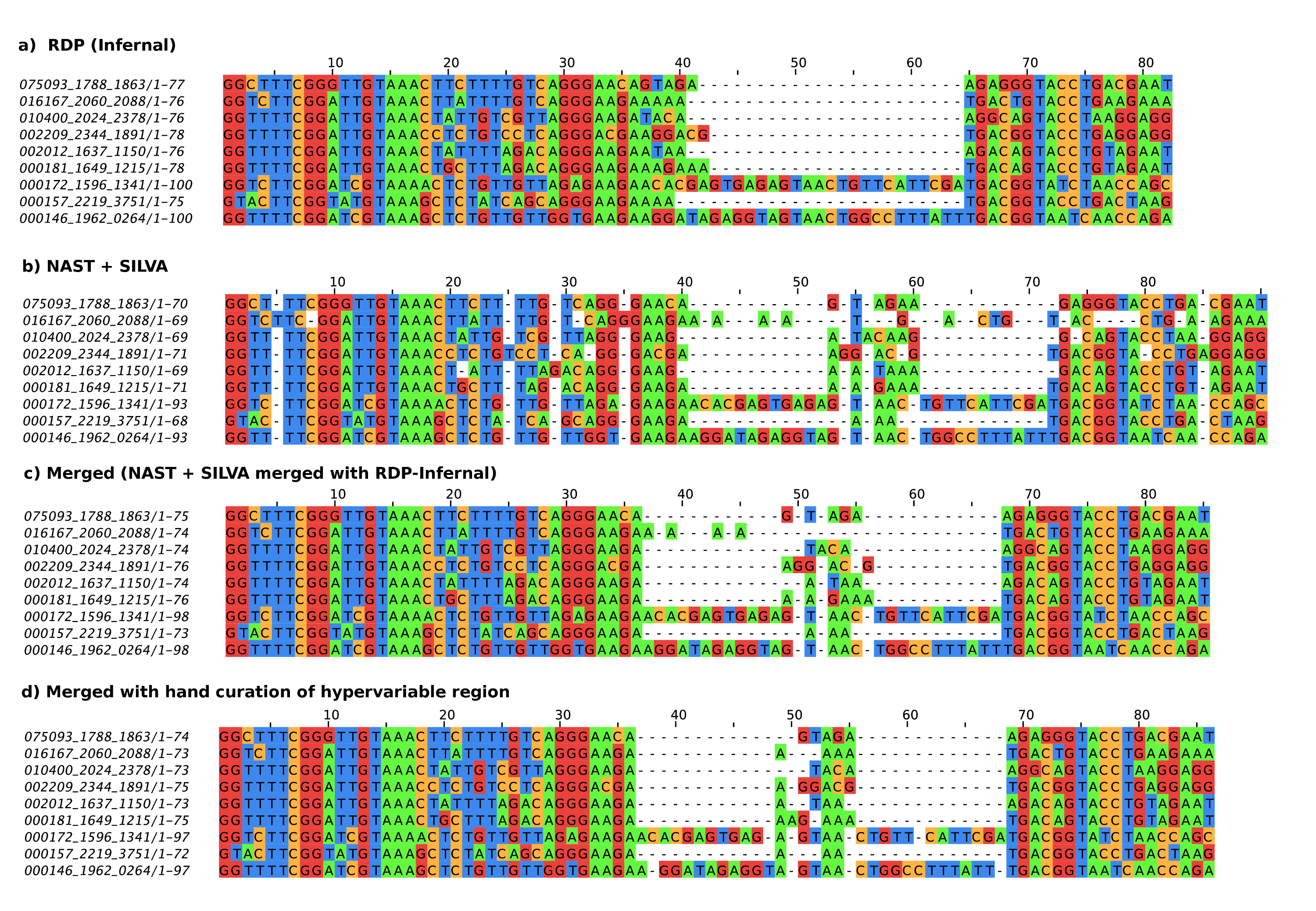

Supplement: Figure S2 — Snippets of 9 reads aligned using the 4 different methods described in this paper. The 9 reads are of the V3 region of the 16S rRNA. (a) Sequences aligned via RDP [30] which uses the Infernal aligner [33]. Note that the hypervariable region is left unaligned (bases 36 through 64). (b) Sequences aligned via NAST [29] (as implemented by Mothur [11]) to the SILVA [32] database. Notice the inconsistencies in the alignment of the regions with strong secondary structure conservation (bases 5, 25, 29, 72 through 79, and 85). (c) Sequences aligned using the merge program in the tool we developed, TORNADO (http://tornado.igb.uiuc.edu). The merge process takes the unaligned, hypervariable parts of the sequence aligned by (a) and replaces them by the alignment in (b). (d) Sequences aligned like in (c), but with the final hand-curation step of the hypervariable regions. (TIFF) [file pone.0015220.s002.tiff]

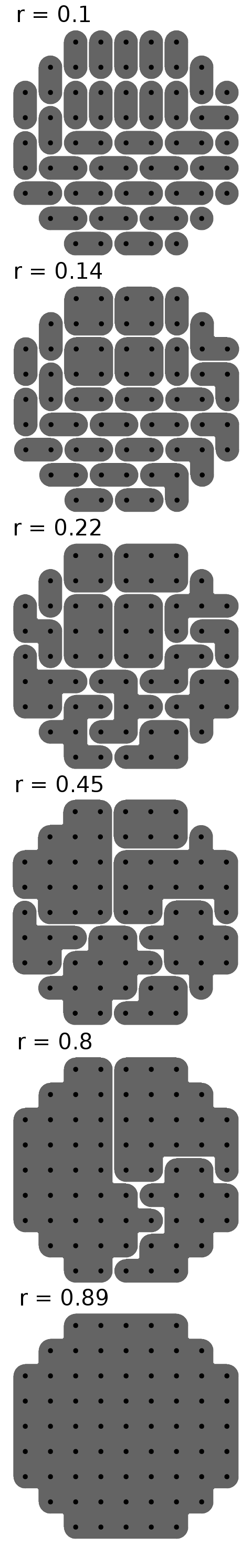

Supplement: Figure S3 — Illustration of the process of the complete linkage algorithm. Smaller clusters are progressively merged into larger ones as long as no two elements of a cluster are farther than from each other. (TIFF) [file pone.0015220.s003.tiff]

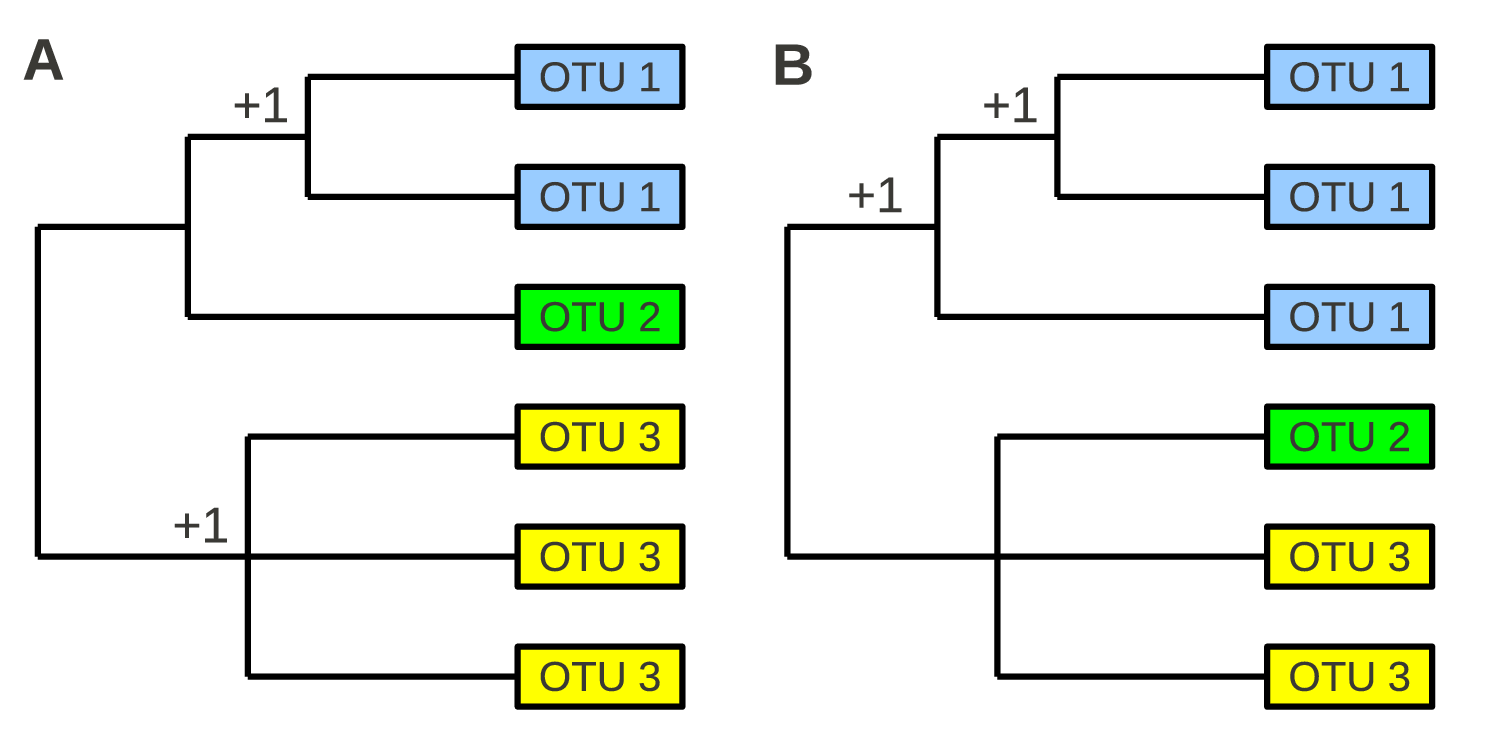

Supplement: Figure S4 — Sketch of the calculation of the number of clades with uniform OTUs. A phylogenetic tree with 2 different cluster (OTU) assignments is shown. The cluster assignment is indicated by OTU number and color. Both cluster assignments have 2 uniform clades (interior nodes indicated by +1). (a) The uniform clades are: one made up of two OTU 1 organisms, and one made up of three OTU 3 organisms. (b) The uniform clades are: one made up of two OTU 1 organisms and one made up of three OTU 1 organisms. (TIFF) [file pone.0015220.s004.tiff]

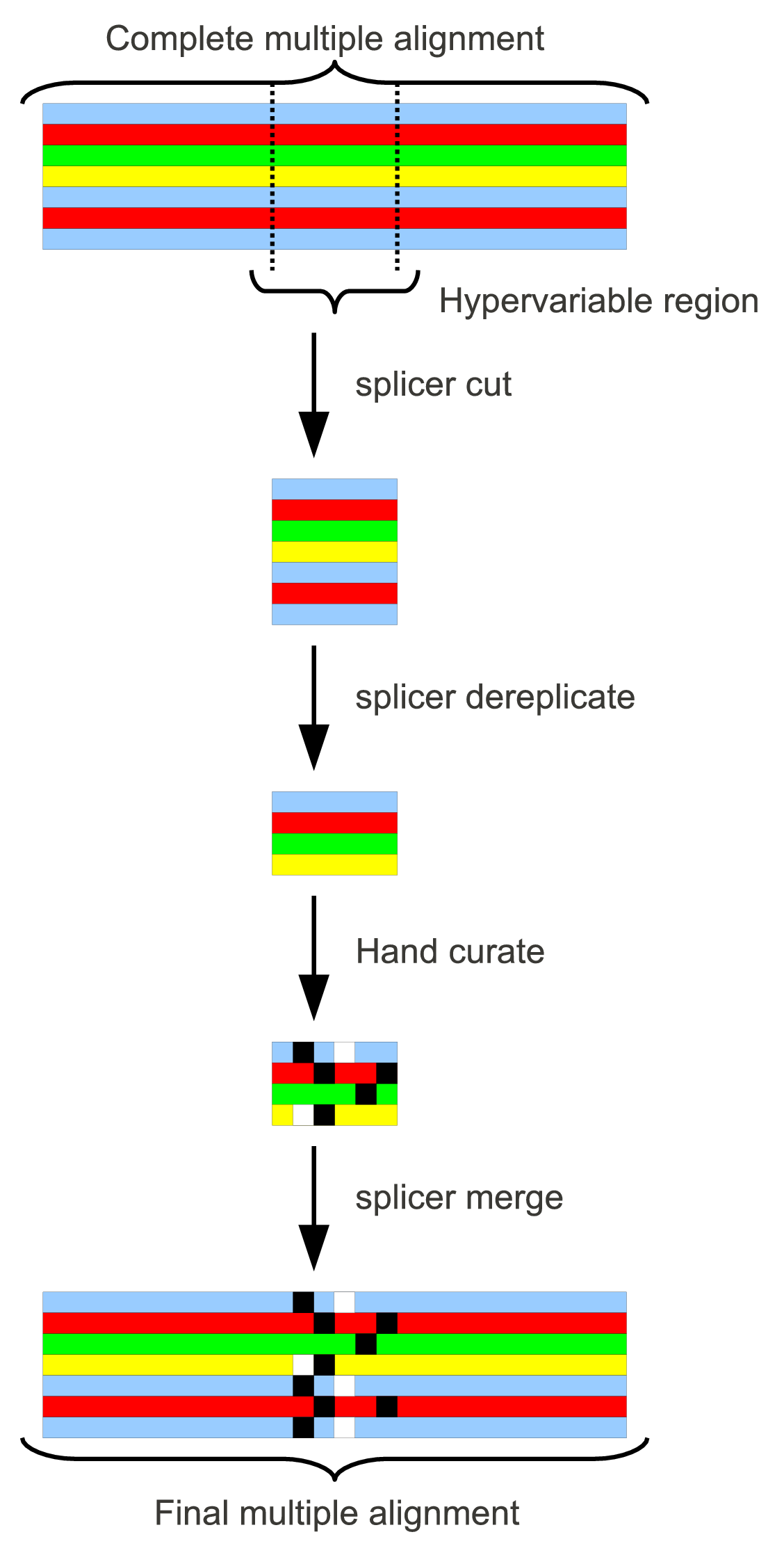

Supplement: Figure S5 — Using splicer, a part of the TORNADO pipeline, to perform hand curation. Dereplicating the hypervariable region significantly reduces the effective number of snippets of sequences one needs to hand curate (4 instead of 6 in this example). In our dataset of around 20,000 sequences, there were only around 200 unique sequence snippets in the hypervariable region varying in length between 1 and 30 bp. (TIFF) [file pone.0015220.s005.tiff]

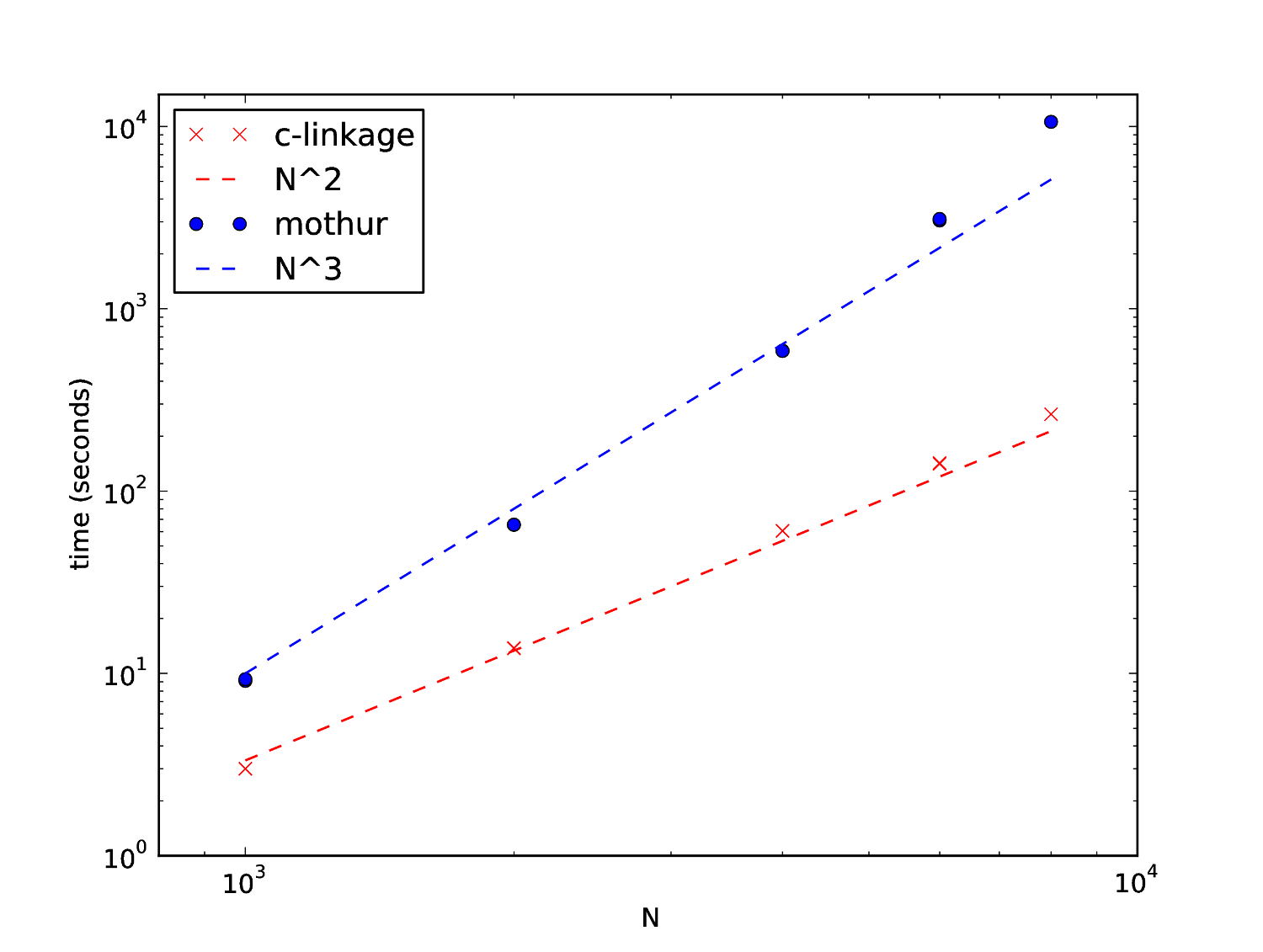

Supplement: Figure S6 — Comparison of running times of c-linkage with the running times of Mothur. The two programs were benchmarked on artificial datasets of 1000, 2000, 4000, 6000 and 8000 elements. The sripts used to generate these datasets and run the benchmarks are available at http://tornado.igb.uiuc.edu. (TIFF) [file pone.0015220.s006.tiff]
